# Feasibility of rigid 3D image registration of high-resolution peripheral quantitative computed tomography images of healing distal radius fractures

**DOI:** 10.1371/journal.pone.0179413

**Published:** 2017-07-25

**Authors:** Joost J. A. de Jong, Patrik Christen, Ryan M. Plett, Roland Chapurlat, Piet P. Geusens, Joop P. W. van den Bergh, Ralph Müller, Bert van Rietbergen

**Affiliations:** 1 NUTRIM School for Nutrition and Translational Research in Metabolism, Maastricht University Medical Center, Maastricht, The Netherlands; 2 Department of Rheumatology, Maastricht University Medical Center, Maastricht, The Netherlands; 3 Institute for Biomechanics, ETH Zurich, Zurich, Switzerland; 4 INSERM French National Institute of Health and Medical Research, University of Lyon, Lyon, France; 5 CAPHRI School for Public Health and Primary Care, Maastricht University Medical Center, Maastricht, The Netherlands; 6 Faculty of Medicine and Life Sciences, Hasselt University, Hasselt, Belgium; 7 Department of Internal Medicine, VieCuri Medical Center, Venlo, The Netherlands; 8 Faculty of Biomedical Engineering, Section Orthopaedic Biomechanics, Eindhoven University of Technology, Eindhoven, The Netherlands; Chongqing University, CHINA

## Abstract

For accurate analysis of bone formation and resorption during fracture healing, correct registration of follow-up onto baseline image is required. A per-fragment approach could improve alignment compared to standard registration based on the whole fractured region. In this exploratory study, we tested the effect of fragment size and displacement on a per-fragment registration, and compared the results of this per-fragment registration to the results of the standard registration in two stable fractures and one unstable fracture.

To test the effect of fragment size and displacement, high-resolution peripheral quantitative computed tomography (HR-pQCT) scans of three unfractured radii were divided into subvolumes. Different displacements in x-, y, or z-direction or rotations around each axis were applied, and each subvolume was registered onto the initial volume to realign it. Next, registration of follow-up onto baseline scan was performed in two stable and one unstable fracture. After coarsely aligning the follow-up onto the baseline scan, a more accurate registration was performed of the whole fracture, i.e. the standard registration, and of each fracture fragment separately, i.e. per-fragment registration. Alignment was checked using overlay images showing baseline, follow-up and overlap between these scans, and by comparing correlation coefficients between the standard and per-fragment registration.

Generally, subvolumes as small as 300 mm^3^ that were displaced up to 0.82 mm in x- or y-, or up to 1.64 mm in z-direction could be realigned correctly. For the fragments of all fractures, correlation coefficients were higher after per-fragment registration compared to standard registration. Most improvement was found in the unstable fracture and one fragment of the unstable fracture did not align correctly.

This exploratory study showed that image registration of individual subvolumes, such as fracture fragments, is feasible in both stable and unstable fractures, and leads to better alignment of these fragments compared to an approach that is based on registration using the whole fractured region. This result is promising for additional analysis of bone formation and resorption in HR-pQCT studies on fracture healing.

## Introduction

In medical image analysis, image registration is a commonly used method where two (or more) images are aligned by optimizing a similarity measure, which is often based on voxel intensity cross-correlation [1] or mutual information. [2, 3] In this way, it is possible to gain valuable information that is conveyed in more than one image, e.g. images from different time points, different modalities or distinct viewpoints. [4] Usually, one image is called the ‘fixed’ or reference image, which is the image on which the second image, called the ‘moving’ image, is registered.

Image registration procedures have been developed for almost every tissue and organ. For bone, image registration has been used for various purposes, including improving the reproducibility of high-resolution computed tomography (CT) derived bone parameters [5], strain mapping of loaded bone [6], and for the analysis of sites of bone formation and resorption at the tibia [7] and finger joints.[8] For fractured bone, Lynch et al. applied image registration to longitudinal CT images of healing distal radius fractures to assess changes in CT image intensity within the fracture gap during the healing process[9], and Tassani et al. used image registration to automatically detect the fracture zone in microCT images of trabecular bone. [10] In the aforementioned studies, mostly a rigid image registration procedure was used because bone is a rigid tissue. [5, 7–10] Zwahlen et al., however, used a deformable (non-rigid) image registration approach because of local deformation of individual trabeculae. [6]

**Fig 1:**
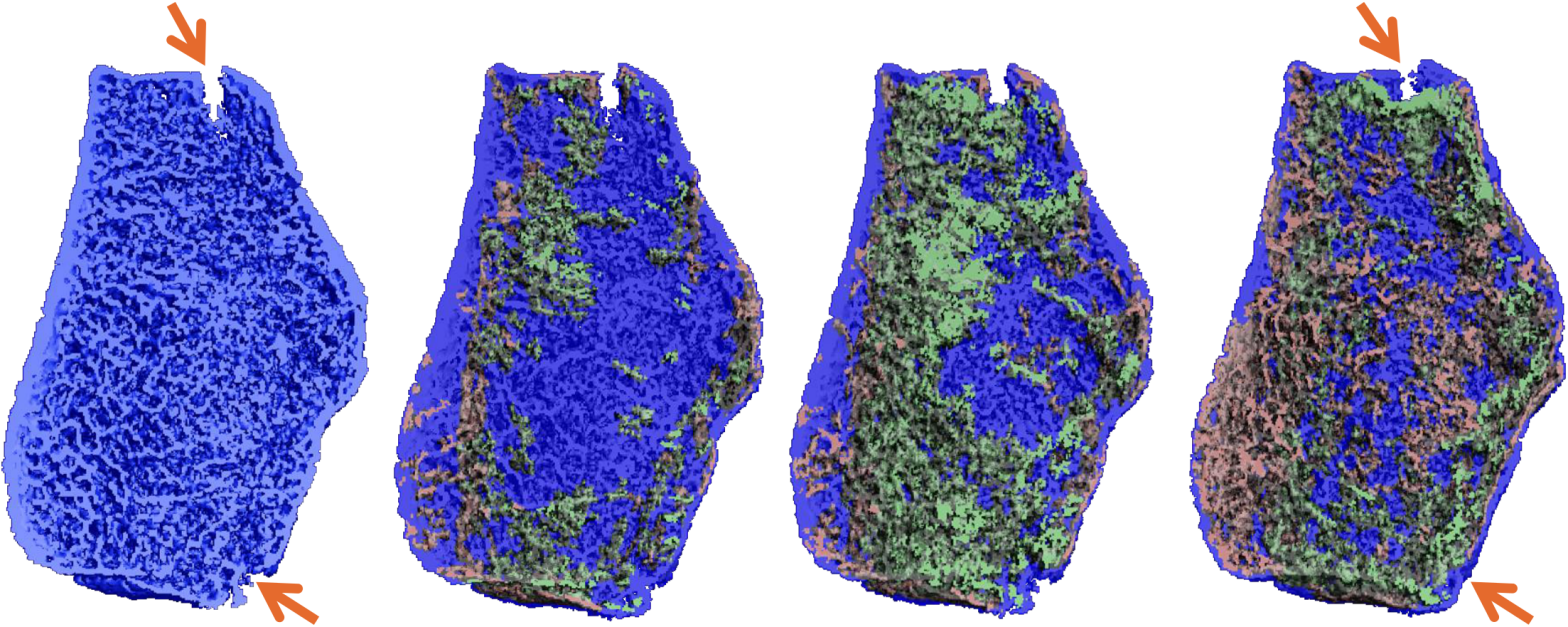
Sites of bone formation and resorption during healing of a stable distal radius fracture. 3D HR-pQCT images of a stable, simple distal radius fracture at 9 days (baseline) and at follow-up 26, 44 and 87 days post-fracture showing regions of bone formation (green), resorption (red) and no change (blue). Images were obtained by superposition of the follow-up images over the baseline image. Cortical fracture locations (indicated by arrows) are bridged at 87 days post-fracture.

Compared to clinical CT used by Lynch et al. [9], high resolution peripheral quantitative CT (HR-pQCT) has a higher spatial resolution which allows for a more detailed assessment of the bone changes at the fracture site. We recently used HR-pQCT to study the healing process of distal radius fractures treated by closed reduction and cast immobilization, and found significant changes in volumetric bone mineral density (vBMD), micro-architecture and biomechanical parameters during the healing process. [11, 12] In addition, superposition of these sequential HR-pQCT images using a rigid 3D image registration in a way similar to Christen et al. did in normal bone, would allow the study of changes of bone formation and resorption, which are intense metabolic processes during fracture healing and may be affected by certain anti-osteoporosis drugs. [13] Applied to preliminary data from a pilot study by Bours et al., this method indeed showed interesting results in a simple, stable fracture (Fig 1). [14] However, although all distal radius fractures in our study were treated as stable fractures, some fractures might be less stable than expected and fracture fragments might move relative to each other in between two HR-pQCT scans. This may lead to erroneous results when the HR-pQCT images are superimposed by the rigid 3D registration procedure in order to visualizing bone formation and resorption, because moved fragments may wrongly be qualified as formed or resorbed bone. Although deformable image registration might be able to correct for such relative movement between fragments, the individual fragments are still mainly composed of rigid bone tissue and, hence, a rigid image registration procedure seems more appropriate.

To limit the amount of wrongly qualified formed or resorbed bone in HR-pQCT images of healing distal radius fractures, we propose to, after a pre-registration, select individual fragments in the moving image and register them separately into the fixed image using rigid 3D image registration. This approach, however, raises several questions that need to be addressed. First of all, the separate fragments are smaller than the scanned bone region itself. It is currently unclear to what extend the registration is affected by using only a subvolume of the moving image as input, instead of using the whole bone region. Second, changes related to the fracture healing process will occur which are larger than changes due to aging or medication and thus the similarity between the fragments can be low, even if correctly registered. To address these issues, we conducted a small exploratory study in which we first tested the proposed method using a subset of HR-pQCT images of a reproducibility study. Then, we applied our method to stable fractures that were minimally displaced and to a fracture where secondary displacement had occurred.

## Materials and methods

### Subjects and image data sets

Two datasets **were** used in this study. The first dataset consisted of HR-pQCT scans of 15 healthy individuals with ages ranging from 21 to 47 years and was presented earlier in the reproducibility study of Boutroy et al. [15] This dataset was also previously used by Christen et al. to study the reproducibility of bone apposition and resorption quantification in healthy subjects. [7] In the present study, three HR-pQCT scans of a single timepoint of three different subjects were randomly selected from this dataset to test the effect of size versus displacement on the fracture registration algorithm. Informed consent was obtained from all participants and the reproducibility study was approved by an independent Ethics Committee (Comité Consultatif de Protection des Personnes dans la Recherche Biomédicale de Lyon).

The second dataset consisted of six HR-pQCT scans that were made during the first 12 weeks of fracture healing in three post-menopausal women with a distal radius fracture who were treated by closed reduction and cast immobilization. Image acquisition has been described previously[11], but briefly, an 18 mm-long region covering the fracture was scanned by HR-pQCT at 1–2, 3–4, 6–8 and 12 weeks postfracture using standard *in vivo* settings (tube voltage 60 kVp, tube current 900 μA, integration time 100 ms, 6 μSv effective dose). Using an isotropic voxelsize of 82 μm, each HR-pQCT measurement thus resulted in transverse 220 slices. After testing the fracture registration algorithm, it was applied onto three fractures that were selected from this dataset to test if a registration based on fragments improved the registration in these fragments. Since movement is most likely to occur during the early phases of fracture healing, when the fragments are not stabilized yet, baseline images at 1–2 weeks post-fracture and follow-up images at 3–4 weeks post-fracture were used. Furthermore, HR-pQCT images of the fractures were selected on having no or minimal motion artifacts.[16] Informed consent was obtained from all participants and the fracture healing study was approved by the local Medical Ethics Committee (NTR3821).

### Description of the fracture registration algorithm

For a correct analysis of sites of bone formation and resorption as calculated from two consecutive HR-pQCT scans, a proper registration of the two scans is required. The rigid 3D registration procedure (Image Processing Language version 5.16/regis 1.09b, Scanco Medical AG, Brütissellen, Switzerland) used in the present study can be divided into two parts:

1. A pre-registration to coarsely align the follow-up image onto the baseline image using solid volumes of the periosteal contours of these images
2. Fine registration per fragment to precisely register each fragment from the follow-up image onto the baseline image using the gray-scale images within the contours of the fragments

#### Pre-registration

Because the orientation of the fractured radius may differ between two HR-pQCT scans, a pre-registration was required to coarsely align the follow-up image onto the baseline image. This pre-registration was based on solid volumes within the periosteal contours in the two images. The periosteal contours were derived using the standard semi-automatic contouring method according to the manufacturer’s instructions. [15] Since rotations of the radius around the x- and y-axis are restricted due to fixation of the forearm during scanning procedure, the pre-registration started by rotating the mask of the follow-up image, that has been downscaled 10 times, in steps of 22.5 degrees around the z-axis to save time. The angle at which the best overlap was found was then used as the starting position for a registration at 4 times downscaling in which the follow-up masks was iteratively translated along and rotated around each axis until the best overlap was found.

#### Fine registration per fragment

After this course alignment, a fine 3D rigid registration of the individual fragments in the follow-up image onto the baseline image was performed. The rotations and translations found in the pre-registration were used as starting position for each fragment in the fine registration. Since each fragment is supposed to be already more or less in place, this fine registration was performed using two consecutive registrations at different resolutions: the first registration at one-fourth of the image resolution, i.e. images were downscaled four times, which was then followed by the final registration at the original image resolution. In the fine registration, both the total follow-up and baseline images were used as input images, but only information within the volume of interest (VOI) in each image was counted in calculation of best overlap. The VOI in the moving image was the contoured fragment, and the VOI in the fixed image the region within the periosteal contour.

#### Standard registration

The standard registration used the same procedure at the same settings, i.e. a pre-registration to coarsely align the follow-up image to the baseline image followed by a fine registration. In the fine registration step, the VOI of the moving image consisted of the whole bone region within the periosteal contour, instead of a fragment.

All registrations were performed on a HP Integrity rx2660 system equipped with four cores running at 1.42 GHz and 32 GB RAM.

### Effect of fragment size, offset and step-size

**Fig 2:**
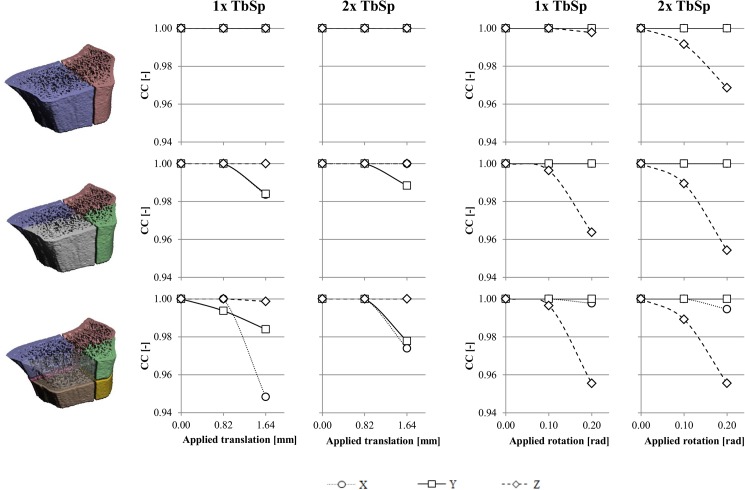
Subvolumes and corresponding correlation coefficients. Average correlation coefficients for registration of volumes with different sizes, using different step-sizes after different applied translations in x-, y- and z-direction and after different applied rotations around the x-, y- and z-axis.

To test how well small fragments of different size can be registered, the 3D volume of each radius scan was divided into 2, 4 and 8 subvolumes (Fig 2). Each subvolume was then shifted from its original position by 10 or 20 voxels (0.82 and 1.64 mm, respectively) in the x-, y-, or z-direction, or rotated around the x-, y-, or z-axis by 0.1 or 0.2 rad (5.7 and 11.5 degrees, respectively). Next, each shifted/rotated subvolume was registered onto the initial, total scan to see if it could be registered correctly. With the registration a simplex search algorithm was used that performs translation/rotation trial steps of a typical size. For periodic structures, such as bone, it was expected that the translation step size should be close to the size of the trabecular separation, to avoid ending at a local minimum. To test the effect of the typical step-size in each iteration, the same procedure described above was repeated for step-sizes of 1 and 2 times the mean trabecular separation per scan. To quantify the agreement at each iteration, a correlation coefficient (CC) was calculated according to Eq 1: [17]

$$cc=\frac{\sum_{i=1}^{nv}v_{i}w_{i}}{\sqrt{\sum_{i=1}^{nv}v_{i}v_{i}\sum_{i=1}^{nv}w_{i}w_{i}}}$$ (Eq 1)

where *v*_*i*_ is the value of voxel *i* in image 1, i.e. the fixed image; *w*_*i*_ the value of a corresponding voxel in image 2, i.e. the moving image, after translation/rotation using tri-linear interpolation; and *nv* the number of voxels in the evaluated region. Since the image information per subvolume is exactly the same as in the initial image, a correlation coefficient of 1.000 indicates a perfect registration.

### Implementation onto healing fractures

When the performance of the registration per part of different sizes, maximum allowed offset and the optimal step-size were known, the method was applied onto several fracture cases: ‘simple’ fractures with two fragments that are relatively stable over time, and ‘complex’ fractures with three or more fragments that have moved relative to each other over time. To be able to study the expected beneficial effect of per-fragment registration, each scan of each fracture case was also registered using the standard registration approach which uses the whole bone region in the follow-up image in the registration. To test the inter-operator reproducibility of the per-fragment registration, each fragment was contoured independently by two experienced operators.

Per fragment the result of the registration was determined qualitatively by checking overlay images. These overlay images were created by combining segmented 3D volumes of the fracture at baseline and the registered follow-up image, with voxels in the baseline volume set to value 1 (red) and in the follow-up volume set to value 2 (green). Thus, after combining these volumes, overlapping regions in the overlay image have value 3 (purple). [18] The segmented 3D volumes were created from the gray-scale images by applying a Gaussian filter (sigma = 0.7, width = 1.0 voxel) to remove noise followed by thresholding (threshold 120 mgHA/cm^3^). Furthermore, correlation coefficients [17] within each fragment region were compared between the registrations based on the standard approach and the per-fragment approach to see whether the registration was improved.

### Statistics

A Wilcoxon signed-rank test was used to compare the correlation coefficients per fragment obtained using the standard registration and per-fragment registration approach.

The inter-operator reproducibility was expressed using the intraclass correlation coefficient (ICC, two-way random effect model) between the correlation coefficients per fragment obtained from the per-fragment registration method using the contours made by the two operators.

All statistical analyses were performed using IBM SPSS Statistics for Windows, version 20.0 (IBM Corp. Armonk, NY, USA).

## Results

### Effect of fragment size, offset and step-size

The average correlation coefficients for registration of the subvolumes of different sizes, using different step-sizes after different applied translations in x-, y- and z-direction and after different applied rotations around the x-, y- and z-axis are presented in Fig 2. The average volumes ± standard deviation (SD) of the one-halve, one-quarter and one-eight parts were 1200 ± 130 mm^3^, 596 ± 108 mm^3^, and 298 ± 74 mm^3^, respectively. The trabecular separation measured in the total volumes was 0.561 mm, 0.626 mm, and 0.673 mm.

Regarding the applied translations, a correct registration was possible after a translation of the one-halve parts of 0.82 and 1.64 mm in each direction, regardless of step-size. For one-quarter and one-eight parts, in general a correct registration was found after translations of 0.82 and 1.64 mm in the z-direction, but not in the x- and y-direction: then the registration failed at translation of 1.64 mm.

Regarding the applied rotations, a correct registration was found for rotations of the one-halve and one-quarter parts around the x- and y-axis by 0.10 and 0.20 radians, regardless of step-size, whereas registration failed after rotations around the z-axis. For one-eight parts, registration was correct for rotations of 0.1 and 0.2 radians around the x-axis and 0.1 radians around the y-axis, but failed for rotations of 0.2 radians around the y-axis and 0.1 and 0.2 radians around the z-axis, regardless of step-size.

### Application to fractures

**Fig 3:**
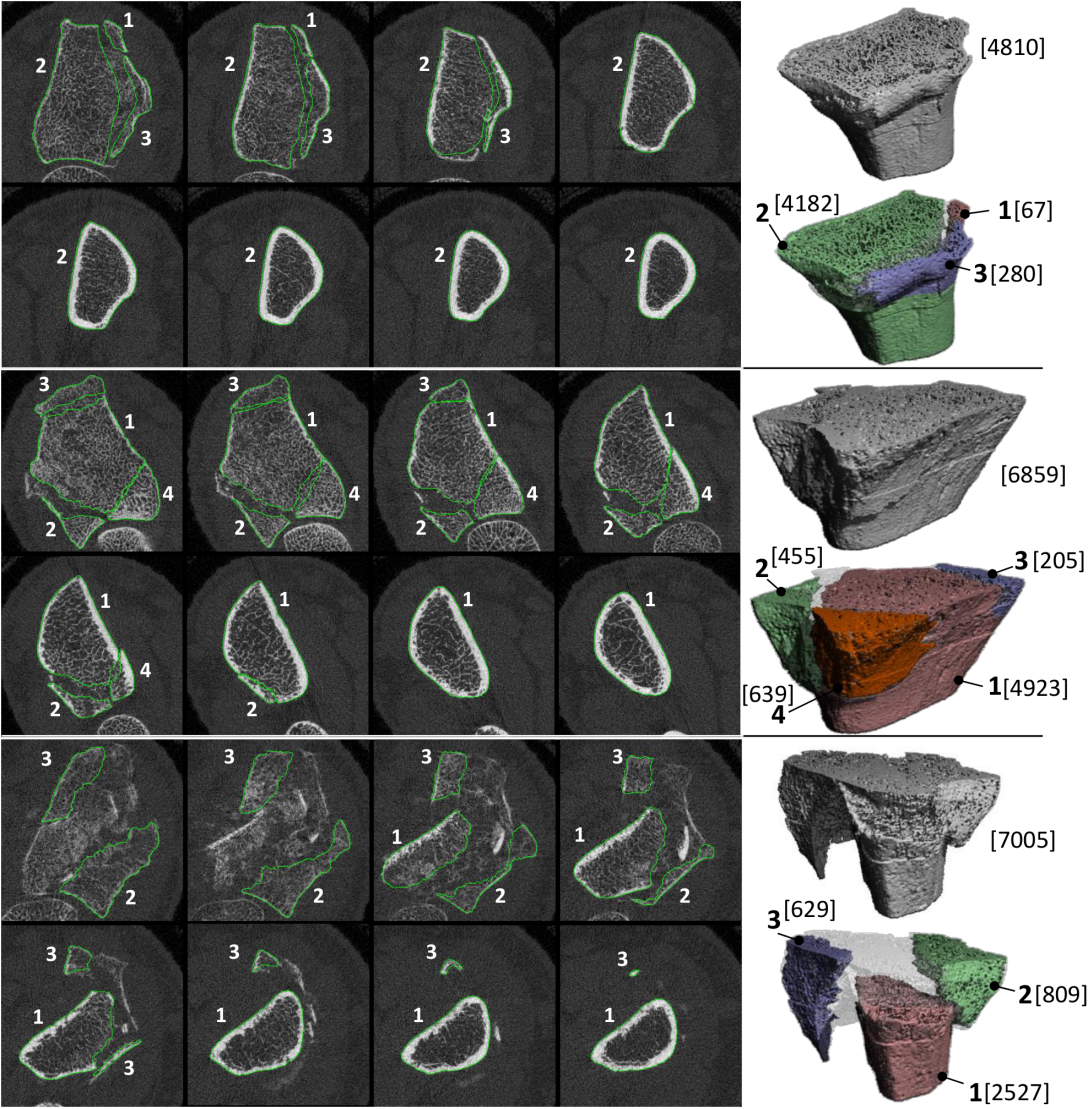
Three fracture cases. 2D HR-pQCT slices with contours (left) and 3D model (right) of the fragments in two stable fractures (top and middle) and a fracture with secondary displacement (bottom) that were registered in this study. The volume [mm^3^] per fragment as well as of the complete fracture is shown between brackets.

In Fig 3, 2D HR-pQCT slices with the contoured fragments are shown, as well as 3D models of each fracture with the fragments visualized in different colors. In each fracture, one large volume could be defined, with volumes ranging from 2527 mm^3^ to 4923 mm^3^ covering 36% to 87%, respectively, of the total volume. Additionally, two or three smaller fragments could be contoured per fracture and their volumes ranged from 67 mm^3^ to 809 mm^3^.

**Fig 4:**
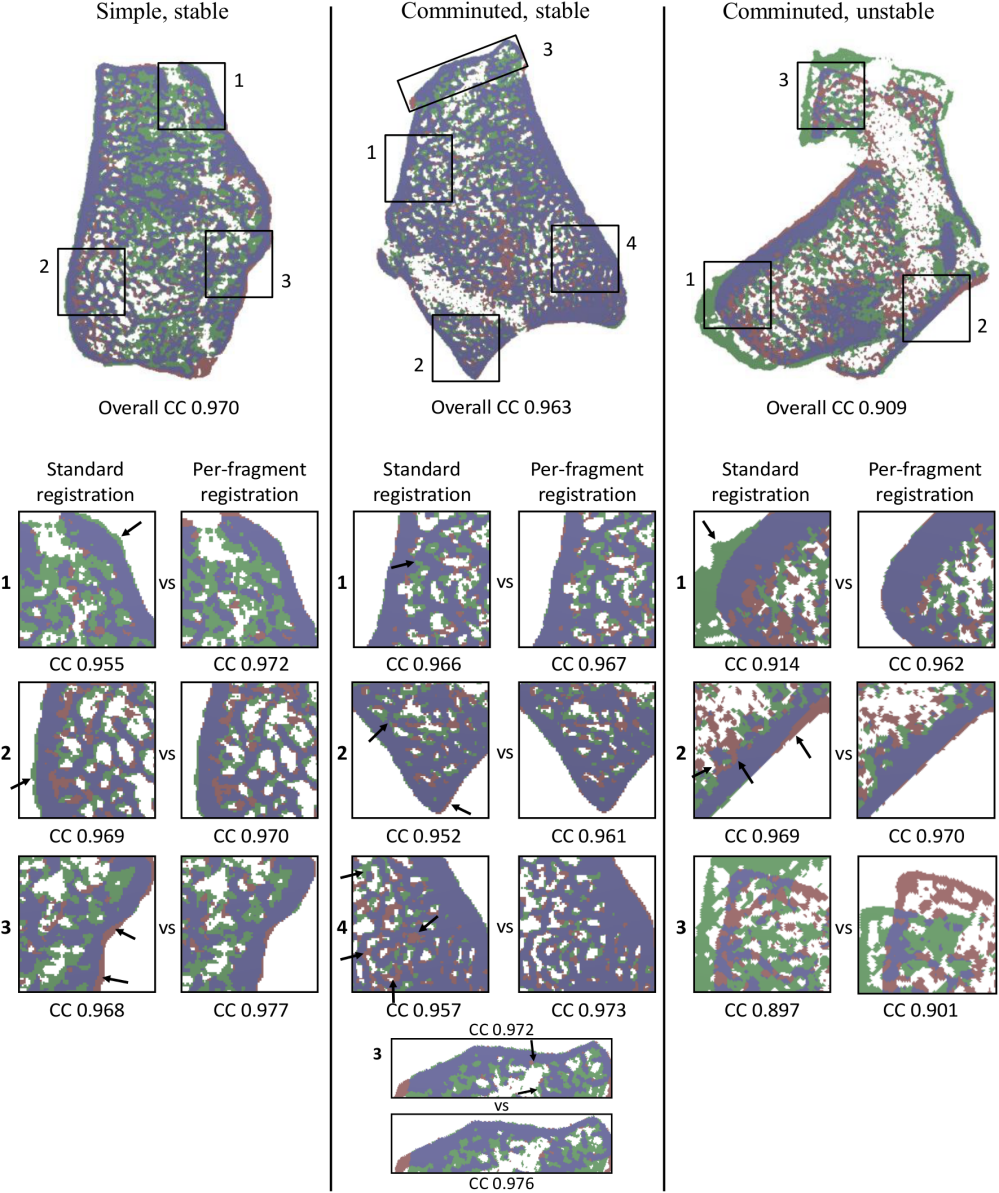
Overlay images of three fracture cases. Overlay image after standard registration of the whole bone region in the follow-up image (3–4 weeks post-fracture) onto baseline (1–2 weeks post-fracture) with corresponding correlation coefficients (top). Overlapping regions (purple) are shown as well as regions belonging to baseline (red) and follow-up (green). To check whether the per-fragment registration improved the results compared to the standard registration, overlay images and corresponding correlation coefficients are presented for the same subregions obtained after standard and per-fragment registration. Arrows indicate locations of clear improvement after per-fragment registration. Correlation coefficients, which are reported below each fragment, are higher after per-fragment registration than after standard registration (Wilcoxon signed-rank test, p = 0.005).

Overlay images and corresponding correlation coefficients resulting from the registration using the whole bone region in the follow-up scan, and from the registration per fragment are shown in Fig 4. The overlay images suggest that registration based on the whole bone region resulted in an (almost) correct registration in the two stable fractures but not for the unstable fracture. Corresponding correlation coefficients were 0.970, 0.963 and 0.909, respectively. Compared to the standard registration using the whole bone region, the per-fragment registration resulted in significantly higher correlation coefficients (p = 0.005), indicating better alignment for these fragments. The largest improvement in correlation coefficient was observed in fragment 1 of the unstable fracture. Fragment 3 of the unstable fracture showed improved correlation coefficient, while the overlay image clearly showed a misalignment of this fragment after per-fragment registration.

The inter-operator reproducibility of the per-fragment registration method was excellent (ICC = 0.994, p < 0.001).

Computational time per fracture for the per-fragment registration method was 7 minutes and 25 seconds on average, whereas the duration of the standard registration method was 4 minutes and 24 seconds per fracture.

## Discussion

In this exploratory study, we showed for the first time that image registration of separate (fracture) fragments is feasible in both stable and unstable fractures, and leads to better alignment of these fragments compared to an approach that is based on registration using the whole bone region.

Not unexpected, correct realignment occurred more for larger parts than for smaller parts. In smaller parts, realigned was observed after an offset in the z-directions and applied rotations in the z-plane. Interestingly, this movement is likely similar to what happens in case of secondary displacement: the distal part of the fracture collapses over the proximal part in the longitudinal direction, with no or relatively little rotation in the transversal plane.

Based on our results, a step-size similar to the trabecular separation can be advised. This finding is not surprising, since most parts consist mainly of trabeculae. With step-sizes smaller than the trabecular separation, the registration might find a local minimum, hence leading to a non-optimal registration. By using a step-size of at least the trabecular separation, the chance of finding a local minimum is probably less likely, but more thorough tests should be performed to find the optimal setting for the step-size. If individual measurement of trabecular separation is not feasible in a practical setting, using a step-size is advised of approximately 0.7 mm, which is the trabecular separation at the distal radius of osteoporotic and osteopenic post-menopausal women. [15]

In the fractured cases, alignment of the fragments was improved in all cases when the fragments were registered separately in comparison to when the whole bone region was used in the registration. The challenge is to determine for which fractures the per-fragment registration is required in order to obtain reliable results on bone apposition and resorption. It is expected that the stable fractures were sufficiently aligned using the standard registration approach to determine the amount of bone formation and resorption since correlation coefficient were already high and no major increases in correlation coefficients were observed after per-fragment registration. In the unstable fracture, however, a major improvement in alignment was observed for fragment 1. This indicates that unstable fractures can benefit most from the per-fragment registration as presented in this study.

On the other hand, per-fragment registration of fragment 3 in the unstable fracture did not result in a satisfactory alignment. An explanation can be found in the size of the fragment with respect to the displacement. Being the smallest fragment in the unstable fracture with a volume of 629 mm^3^, it had to overcome a distance of approximately 1 mm (13 voxels) in the x-direction and 2 mm (24 voxels) in the y-direction. Since the one-halve volumes with a volume of approximately 600 mm^3^ failed to realign correctly after displacements in the x- or- y-direction of > 0.82 mm (10 voxels), this might indicate that the fragment was too small in relation to the displacement.

Although not quantitatively measured, feasible contouring of fracture fragments depends in our experience heavily on the presence and amount of low mineralized callus, the number of fragments, and the degree to which the fragments are interconnected. Therefore, fragments in HR-pQCT scans that were made early post-fracture, i.e. at 1–2 weeks post-fracture, are less difficult to contour than in the HR-pQCT scans made at 3–4 or even 6–8 weeks post-fracture. At 6–8 weeks post-fracture, a clear distinction between the fragments may be severely hindered by the amount of low mineralized callus that has formed at the fracture line. However, this might not be a problem: at the time this low mineralized tissue has formed, the fragments are not likely to move relative to each other as they have already been stabilized by the presence of callus. We therefore expect that only fragments in the scans that are made during the first 4 weeks post-fracture, need contouring.

Double-stack (220 slices / 18 mm) HR-pQCT measurements were made to ensure that most of the fracture region was included. However, even despite including only measurements with no or very limited motion artifacts, a minor shift between the stacks is almost impossible to avoid. Also in our study, this was the case as can be seen by the edges that are visible in the 3D models in Fig 3. To improve alignment of fragments that are more than 110 slices (9 mm) long, it is advised to align the two stacks per measurement before registering volumes from the follow-up scan onto the baseline scan. Another option is to limit the registration to one stack, but this has the disadvantage that fragments may become very small and are thus more likely to end up misaligned.

### Limitations

A few limitations need to be addressed. Although exploratory, we have shown that separate registration of each fragment may improve the total registration in fractures. Future work should therefore focus on an automatic procedure for finding the fracture line and/or contouring different fragments. Second, we did not apply our approach to experimental data where fragment displacement could be controlled. While focusing on *in vivo* patient data, such an experiment would have been beyond the scope of the present study. Furthermore, we only tested one registration approach whereas other image registration algorithms might lead to better results. Last, we only used HR-pQCT scans of good quality. It is therefore unknown to what extent the registration is affected by the presence of motion artifacts.

## Conclusion

This exploratory study showed that image registration of individual (fracture) fragments is feasible in both stable and unstable fractures, and leads to better alignment of these fragments compared to an approach that is based on registration using the whole fractured region. This result is promising for additional analysis of bone resorption and formation based on longitudinal HR-pQCT images of healing fractures, for example in studies on the metabolic effects of anti-osteoporosis medication.

## Supporting information

S1 File SPSS dataset. Correlation coefficients and calculation times of the standard and per-fragment registration approach for each fragment per subject.
(SAV)

S2 File SPSS syntax. Wilcoxon signed-rank test and intraclass correlation coefficient.
(SPS)
